# Research in progress: Medical Research Council United Kingdom Refractory Asthma Stratification Programme (RASP-UK)

**DOI:** 10.1136/thoraxjnl-2015-207326

**Published:** 2015-07-23

**Authors:** Liam G Heaney, Ratko Djukanovic, Ashley Woodcock, Samantha Walker, John G Matthews, Ian D Pavord, Peter Bradding, Robert Niven, Chris E Brightling, Rekha Chaudhuri, Joseph R Arron, David F Choy, Douglas Cowan, Adel Mansur, Andrew Menzies-Gow, Ian Adcock, Kian F Chung, Chris Corrigan, Peter Coyle, Timothy Harrison, Sebastian Johnston, Peter Howarth, James Lordan, Ian Sabroe, Jeannette Bigler, Dirk Smith, Matthew Catley, Richard May, Lisa Pierre, Chris Stevenson, Glenn Crater, Frank Keane, Richard W Costello, Val Hudson, David Supple, Tim Hardman

**Affiliations:** 1 Centre of Infection and Immunity, The Queens's University of Belfast, Belfast, UK; 2 Academic Unit of Clinical and Experimental Sciences, University of Southampton, Southampton, UK; 3 Institute of Inflammation and Repair, The University of Manchester, Manchester Academic Health Science Centre, Manchester, UK; 4 Asthma UK, London, UK; 5 Genentech Inc., South San Francisco, California, USA; 6 Nuffield Department of Medicine, The University of Oxford, Oxford, UK; 7 Department of Infection, Immunity and Inflammation, Institute for Lung Health, University of Leicester, Leicester, UK; 8 Greater Glasgow Health Board, Glasgow, UK; 9 Heart of England NHS Foundation Trust, Birmingham, UK; 10 Royal Brompton & Harefield NHS Foundation Trust, London, UK; 11 Imperial College of Science, Technology and Medicine, London, UK; 12 Kings College London, London, UK; 13 University of Nottingham, Nottingham, UK; 14 The Newcastle upon Tyne NHS Foundation Trust, Newcastle upon Tyne, UK; 15 School of Medicine and Biomedical Sciences, University of Sheffield, Sheffield, UK; 16 Amgen Inc., Thousand Oaks, California, USA; 17 Astra Zeneca, London, UK; 18 Jannsen Research & Development LLC, London, UK; 19 Aerocrine AB, Solna, Sweden; 20 Vitalograph Inc, Ennis, Ireland; 21 Department of Medicine, Royal College of Surgeons Ireland, Dublin, Ireland; 22 Public and Patient Representative, London, UK; 23 Niche Science & Technology, Richmond, UK

**Keywords:** Asthma Mechanisms

## Abstract

The UK Refractory Asthma Stratification Programme (RASP-UK) will explore novel biomarker stratification strategies in severe asthma to improve clinical management and accelerate development of new therapies. Prior asthma mechanistic studies have not stratified on inflammatory phenotype and the understanding of pathophysiological mechanisms in asthma without Type 2 cytokine inflammation is limited. RASP-UK will objectively assess adherence to corticosteroids (CS) and examine a novel composite biomarker strategy to optimise CS dose; this will also address what proportion of patients with severe asthma have persistent symptoms without eosinophilic airways inflammation after progressive CS withdrawal. There will be interactive partnership with the pharmaceutical industry to facilitate access to stratified populations for novel therapeutic studies.

## Introduction

The Medical Research Council has recently funded an ambitious stratified medicines programme in severe asthma (the UK Refractory Asthma Stratification Programme (RASP-UK http://www.rasp.org.uk/). In asthma, disease stratification has traditionally followed a ‘one-size fits all’ approach, based on level of asthma control and response to ‘stepwise’ increases in corticosteroid (CS) treatment. More recently, stratification has been based on inflammatory biomarkers, with sputum analysis and transcriptomic profiling demonstrating that ∼50% of patients with asthma have typical eosinophilic airway inflammation driven by ‘Type 2’ cytokines (T2 cytokines: interleukin (IL)-4, IL-5, IL-13); this T2-high asthma has been shown to respond well to CS therapy. In the remainder of patients, there is limited evidence for a major role for T2 cytokines and this has been termed ‘T2-low asthma’, where patients show little or no response to CS therapy.^1^ There is also evidence of significant disease heterogeneity in severe asthma, with only 25%–50% of patients having the prototypic T2 gene signature consistent with T2-high disease. In many cases of severe asthma, it therefore seems likely that the CS dose has been escalated inappropriately to try and manage persistent symptoms that are not CS responsive. Given the clear evidence that CS responsiveness is confined to T2-high disease, the cornerstone of the RASP-UK stratification programme is around failure of response to CS therapy in severe asthma.

We propose that in patients with severe asthma, failure to respond to high-dose CS treatment occurs for three principal reasons:

1. Non-adherence to CS treatment.
2. Impaired CS responsiveness—in some patients, despite adherence with high-dose inhaled CS, there is persistent T2-high inflammation/eosinophilia and these patients frequently require systemic CS to achieve an acceptable level of disease control.
3. Non-responsiveness to CS in patients with T2-low asthma—we believe that CS will have been escalated inappropriately in this group in clinical practice.

## Non-adherence to CS treatment

Non-adherence to inhaled CS and oral CS therapy is a major clinical problem, present in 30%–50% of patients with difficult-to-control asthma. Non-adherence can be intentional or non-intentional, is poorly detected by physicians and asthma nurses and is associated with poor asthma outcomes and increased healthcare costs.^2^ Moreover, enrolment of non-adherent patients into clinical trials is believed to contribute to the frequently observed improvement in placebo arms of asthma clinical trials, complicating the interpretation of trial results and reducing statistical power. Importantly, expensive new biological therapies targeting T2-high patients can potentially be used inappropriately in patients who would often be controlled with improved adherence to inhaled CS. Patients with difficult-to-control asthma and fractional exhaled nitric oxide (FeNO) >45 ppb have a high risk of exacerbation^3^ and the key clinical question is whether failure to control their disease is because of non-adherence to inhaled CS treatment or whether they have relative CS-resistant T2-high asthma and, thus, require additional treatment with drugs that target T2 mechanisms (eg, anti-IL-5 and anti-IL-13 treatments). RASP-UK will therefore use biomarker-based assessments of inhaled CS exposure and response in biomarker-high patients (‘FeNO suppression test’—reference 4 online supplementary data) using remote monitoring technologies to define CS responsivity and thus optimise inhaled CS where appropriate.

## Impaired CS responsiveness

In some patients with severe asthma, despite adherence with high-dose inhaled CS, there is persistent T2-high inflammation and airway eosinophilia and these patients frequently require systemic CS to achieve disease control. The availability of many new biological therapies targeting T2-high disease in the next 5 years will generate many interesting questions, including differential efficacy between monoclonal antibodies targeting IL-5/IL-5R (mepolizumab, bendralizumab, relizumab), IL-13 (lebrikizumab, tralokinumab) and IL-4Rα (dupilumab). Other strategies targeting the T2-axis, including anti-CrTh2 and novel anti-IgE therapies (quilizumab, Medi-4212, QGE031), will also be targeting overlapping patient groups; identifying which patients respond better to different classes of drugs may require ‘head-to-head’ studies. Many of these new therapies are expected to come to market with a companion diagnostic or predictive biomarker(s) of clinical response.

## Non-responsiveness to CS

Inhaled CS remain the mainstay of asthma treatment, controlling disease through suppression of T2/eosinophilic inflammation in the airway. However, not all symptoms in severe asthma respond to CS, and CS responsiveness is minimal in the absence of T2 inflammation.^1^ Therefore, new algorithms for guiding CS treatment in patients with severe asthma are needed, which are not solely based on clinical symptoms and lung function and which can be delivered in the clinic. Titrating CS against sputum eosinophilia improves asthma control by reducing asthma exacerbations^4^ but this strategy is difficult to deliver in real-world clinical settings. FeNO is straightforward to measure, but when used as a single biomarker, it has been disappointing in titrating CS in patients with moderate asthma.^5^ Periostin is a secreted matricellular protein from a number of tissues including airway epithelial cells and is upregulated by T2 cytokines (IL-4 and IL-13) and is associated with airway eosinophilia in patients with severe asthma.^1^ An assay for serum periostin is being developed to help identify a periostin-high responder population for the anti-IL-13 monoclonal antibody lebrikizumab.

We have previously examined the predictive value of FeNO, blood eosinophils and serum periostin as a composite biomarker to predict exacerbation risk in the placebo arms of clinical trials with lebrikizumab and omalizumab in patients taking at least 500 μg fluticasone propionate and a second controller (see online supplementary data). This analysis has demonstrated that, individually, these biomarkers are all correlated with exacerbation risk, but using the three biomarkers in a ‘composite’ scoring system further differentiated subjects on the basis of exacerbation rate (see online supplementary figure E1). This predictive composite score is independent of asthma symptoms (measured by asthma control questionnaire) and lung function (see online supplementary figures E2 and E3) which current asthma guidelines advocate for treatment adjustment.

RASP-UK will therefore use this scoring system to adjust CS treatment in a randomised, pragmatic, multicentre, parallel group trial in patients with severe asthma and baseline FeNO <45 ppb comparing standard care with treatment guided by composite biomarkers (as discussed above, patients with FeNO ≥45 ppb have a high risk of exacerbation and are not candidates for CS reduction). Exploratory outcomes will examine the prognostic value of selected, novel blood and urine biomarkers from the Unbiased BIOmarkers in PREDiction of respiratory disease outcomes (U-BIOPRED) programme for exacerbation risk (http://www.europeanlung.org/en/projects-and-research/projects/u-biopred/home). The programme will also characterise the inflammatory and microbiomic profiles (viral, bacterial, fungal) in exacerbations in both study arms.

One of the anticipated outcomes of this biomarker-based stratification will be identification of a patient group with T2-low severe asthma who have persistent symptoms and exacerbations which are not CS responsive and in whom T2 inflammation biomarkers (eosinophils, FeNO, periostin) are adequately controlled with moderate doses of inhaled CS. We know that this patient group exists based on data from a comparable sputum stratification study where one-third of patients had a sputum eosinophil count of <1.9% when repeatedly measured over 12 months despite steroid withdrawal.^4^ There are currently few treatment options for these patients representing a significant unmet medical need where new targets need to be identified so drugs can be developed. One of the core aims of RASP-UK is to characterise this group in detail to define important structure/function/symptom relationships. This will include transcriptomic and microbiomic profiling compared with age and gender matched patients with refractory eosinophilic asthma and stable asthma controlled with low-dose inhaled CS. These groups will be followed up for 12 months to identify their exacerbation pattern and stability of the phenotype. Previous research has focused on eosinophilia as a prognostic for exacerbation, but ‘non-eosinophilic cohorts’ also experience exacerbations and potent anti-eosinophilic therapies have not eradicated severe exacerbations in clinical trials. A better understanding of the mechanism and phenotype of exacerbations in this T2-low cohort is fundamental to targeting exacerbations in this group.

## Conclusion

**Figure 1:**
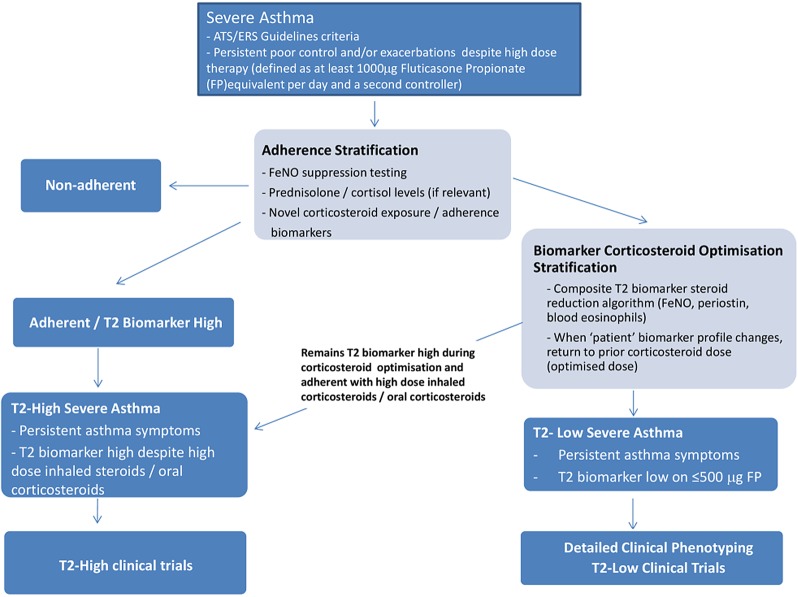
Flow diagram for the RASP-UK Clinical Stratification Programme. ATS, American Thoracic Society; ERS, European Respiratory Society; FeNO, fractional exhaled nitric oxide; FP, Fluticasone Propionate; RASP-UK, UK Refractory Asthma Stratification Programme.

RASP-UK is a logical, clinically focused, stratification programme which will develop a clinical model to assess and phenotype patients with severe asthma (figure 1). It will identify appropriate patients for novel biological treatments that target the T2-cytokine axis (T2 biomarker high when adherent to high dose inhaled CS), will optimise CS treatment so that excessive CS exposure does not cause unnecessary morbidity and will facilitate a research focus and better understanding in a major area of ‘unmet need’, specifically non-T2/non-CS responsive severe asthma. Harmonised protocols for patient phenotyping will also allow stratified patient groups to enter proof of concept clinical trials which target the biology of their disease rather than using the usual broad inclusion criteria, where many of the patients are unlikely to respond; this will streamline and reduce size for early drug development in severe asthma. The RASP-UK Consortium vision is to effect a paradigm shift in asthma care through a logical stratified approach to severe asthma and to establish a long-term collaborative infrastructure for translational research excellence in the UK.

## Supplementary Material

Web supplement
