# Supplementary material for: Research in progress: Medical Research Council United Kingdom Refractory Asthma Stratification Programme (RASP-UK)
Source: Thorax. 2015 Jul 23;71(2):187–9. doi: 10.1136/thoraxjnl-2015-207326 (PMC4752622; doi:10.1136/thoraxjnl-2015-207326)

Research in Progress –Medical Research Council Refractory Asthma Stratification Consortium – Online Supplement

Liam G Heaney[1], Ratko Djukanovic[2], Ashley Woodcock[3], Samantha Walker[4], [5]John G. Matthews[5], Ian D Pavord[6], Peter Bradding[7], Robert Niven[3], Chris E Brightling[7], Rekha Chaudhuri[8], Joseph R. Arron[5], David F. Choy[5], Douglas Cowan[8], Adel Mansur[9], Andrew Menzies-Gow[10], Ian Adcock[11], Kian F Chung[11], Chris Corrigan[12], Peter Coyle[1], Timothy Harrison[13], Sebastian Johnston[11], Peter Howarth[2], James Lordan[14], Ian Sabroe[15], Jeannette Bigler[16], Dirk Smith[16], Matthew Cattley[17], Richard May[17], Lisa Pierre[18], Chris Stevenson[18], Glenn Crater[19], Frank Keane[20], Costello RW[21], Val Hudson[22], David Supple[22], Tim Hardman[23].

RASP-UK Consortium Partners – [1]The Queens's University of Belfast, [2]University of Southampton, [3]The University of Manchester, [4]Asthma UK, [5]Genentech Inc., [6]The University of Oxford, [7]University of Leicester, [8]Greater Glasgow Health Board, [9]Heart of England NHS Foundation Trust, [10]Royal Brompton & Harefield NHS Foundation Trust, [11]Imperial College of Science, Technology and Medicine, [12]Kings College London, [13]University of Nottingham,[14]The Newcastle upon Tyne NHS Foundation Trust, [15]University of Sheffield, , [16]Amgen Inc., [17]Astra Zeneca , [18]Jannsen Research & Development LLC, [19]Aerocrine AB, [20]Vitalograph, [21]Royal College of Surgeons Ireland, [22]Public and Patient Representative, [23]Niche Science & Technology.

Address for Correspondence:

Professor Liam Heaney

Centre for Infection and Immunity, Queen's University of Belfast,

Level 8, Belfast City Hospital,

Lisburn Road, Belfast,

Northern Ireland, UK.

BT9 7AB.

Telephone: ++44 (0)28 90 26 3821

Fax: ++44 (0)28 90 26 3879

Email: l.heaney@qub.ac.uk

Non-adherence to corticosteroid treatment

Non-adherence to medication in asthma common, yet poorly detected by physicians, and is associated with poor asthma outcomes and increased healthcare costs (1,2,3). To maximise benefits to patients and provide cost-effective treatment strategies, it is essential to develop objective biomarker strategies to confirm whether patients are adhering to their inhaled corticosteroids or whether they have corticosteroid-unresponsive inflammation.

Our Consortium has demonstrated that the fall in fractional exhaled nitric oxide (FeNO) from baseline after a directly observed inhaled corticosteroid (ICS) challenge (daily for 7 days) distinguishes asthmatic patients with steroid-insensitive T2-High asthma, (where FeNO levels remain high despite ICS treatment), from those who are non-adherent with ICS, in whom a marked fall in FeNO (>40% from baseline) occurs with directly observed treatment (4). We have has thus defined a functional biomarker test ("FeNO suppression test") of ICS exposure in patients with asthma with an initial FeNO >45ppb. The RASP-UK programme will combine the use remote monitoring technology (Aerocrine NIOX MINO Data Management Program) plus Smart inhaler technology (Vitalograph INCA device, see reference 5) to monitor concurrent FeNO and ICS use.

Non-responsiveness to corticosteroids

We have previously examined the predictive value of using FeNO, blood eosinophils and periostin as a composite biomarker to predict exacerbation risk in the placebo arms of clinical trials conducted with lebrikizumab and with omalizumab in patients taking at least 500 μg fluticasone propionate and a second controller (6,7,8). This analysis has demonstrated that:

- individually, all of these biomarkers are correlated with exacerbation risk, but including the three biomarkers in a “composite” scoring system further differentiated subjects on the basis of exacerbation rate (figure E1);

- the composite score is calculated from the individual biomarkers as the average of all three biomarker scores, rounded to the nearest integer, to give the "composite score" [score of 0, 1 or 2 – Table E1]. A higher score predicts a significantly greater exacerbation risk, with a score of 2 associated with a 2-fold higher risk of exacerbation compared with the minimum score of 0;

- the composite score is independent of asthma symptoms (as assessed by asthma control questionnaire [ACQ, figure E2] and lung function (FEV_1, Figure E3) which current asthma guidelines advocate for treatment adjustment;

- the composite scoring system allows identification of a ‘low-risk’ patients with a low exacerbation rate where we believe we can safely reduce corticosteroid dose in this group

Using this straightforward biomarker score to adjust corticosteroids, RASP-UK will conduct a randomised, pragmatic, multi-centre, parallel group trial in patients with severe asthma (persistent symptoms despite treatment with at least 1000 ug fluticasone propionate) and initial FeNO <45 ppb [as discussed previosuly, patients with FeNO >45 ppb have a high risk of exacerbation (9), so these patients are NOT candidates for corticosteroid reduction]. RASP-UK will compare a composite biomarker based strategy to adjust corticosteroid treatment compared with standard care and the primary outcome will be the proportion of patients with any reduction in ICS or oral CS dose from baseline to Week 48.

The study will also explore the prognostic value of the individual and composite biomarkers in predicting severe exacerbations and examine additional novel blood biomarkers for eosinophilic inflammation identified by the UBIOPRED programme [http://www.europeanlung.org/en/projects-and-research/projects/u-biopred/home]. It will also focus on the inflammatory and microbiomic profiles in patients during exacerbations to confirm biomarker-stratified patients are non-eosinophilic during these events.

RASP-UK will explore potential collaborative engagement with new partners over the duration of the programme. This will be by agreement of the Executive Management Team and the Medical Research Council. For clinical sites, this would require that additional resource be provided from an external funding source.

References

1. Gamble J, Stevenson M, McClean E et al. The prevalence of non-adherence in difficult asthma. Am J Resp Crit Care Med. 2009;180(9):817-22

2. Murphy AC, Proeschal A, Brightling CE et al. The relationship between clinical outcomes and medication adherence in difficult-to-control asthma. Thorax. 2012 Aug;67(8):751-3

3. Heaney LG, Horne R. Non-adherence in difficult asthma: time to take it seriously. Thorax. 2012;67(3):268-70

4. McNicholl DM, Stevenson M, McGarvey LP et al. The utility of fractional exhaled nitric oxide suppression in the identification of non-adherence in difficult asthma. Am J Respir Crit Care Med 2012;186(11):1102-8

5. Holmes MS, Seheult JN, Geraghty C et al. A method of estimating inspiratory flow rate and volume from an inhaler using acoustic measurements. Physiol Meas 2013;34(8):903-14

6. Hanania NA, Alpan O, Hamilos DL et al. Omalizumab in severe allergic asthma inadequately controlled with standard therapy: a randomized trial. Ann Intern Med 2011 154:573

7. Corren J, Lemanske RF, Hanania NA et al. Lebrikizumab treatment in adults with asthma. N Engl J Med. 2011;365(12):1088-98

8. Hanania NA, Noonan M, Corren J et al. Efficacy and Safety of Lebrikizumab in Severe Uncontrolled Asthma: Results from LUTE & VERSE Phase II Trials. Number. AAAAI meeting 2014 Session Number: 5611

9. Kupczyk M, ten Brinke A, Sterk PJ, Bel EH, Papi A, Chanez P, Nizankowska-Mogilnicka E, Gjomarkaj M, Gaga M, Brusselle G, Dahlén B, Dahlén SE; BIOAIR investigators. Frequent exacerbators-a distinct phenotype of severe asthma. Clin Exp Allergy. 2014 Feb;44(2):212-221

Legends to Figures Online Supplement

Figure E1 - A higher composite biomarker score further differentiated subjects on the basis of exacerbation rate (mean, 95% CI), with a composite biomarker score of 2 having a 2-fold higher risk of exacerbation compared to the composite score of 0.

Figure E2 and E3 – The composite biomarker has no relationship with symptoms [figure E2 - Asthma Control Questionnaire, ACQ5] or lung function [figure E3 - FEV1% predicted] which are usually used to up-titrate corticosteroids

Table E1. The composite biomarker score is calculated from the individual biomarker scores and is the AVERAGE of all 3 scores rounded to the nearest integer to give the "composite score" [score of 0, 1 or 2]

**Biomarker Score**	**0**	**1**	**2**
**FeNO (ppb)**	<15	15 - <30	≥30
**Blood eosinophil count (N/μL)**	< 150	150 - <300	≥300
**Periostin (ng/ml)**	<45	45 - <55	≥55

Figure E1

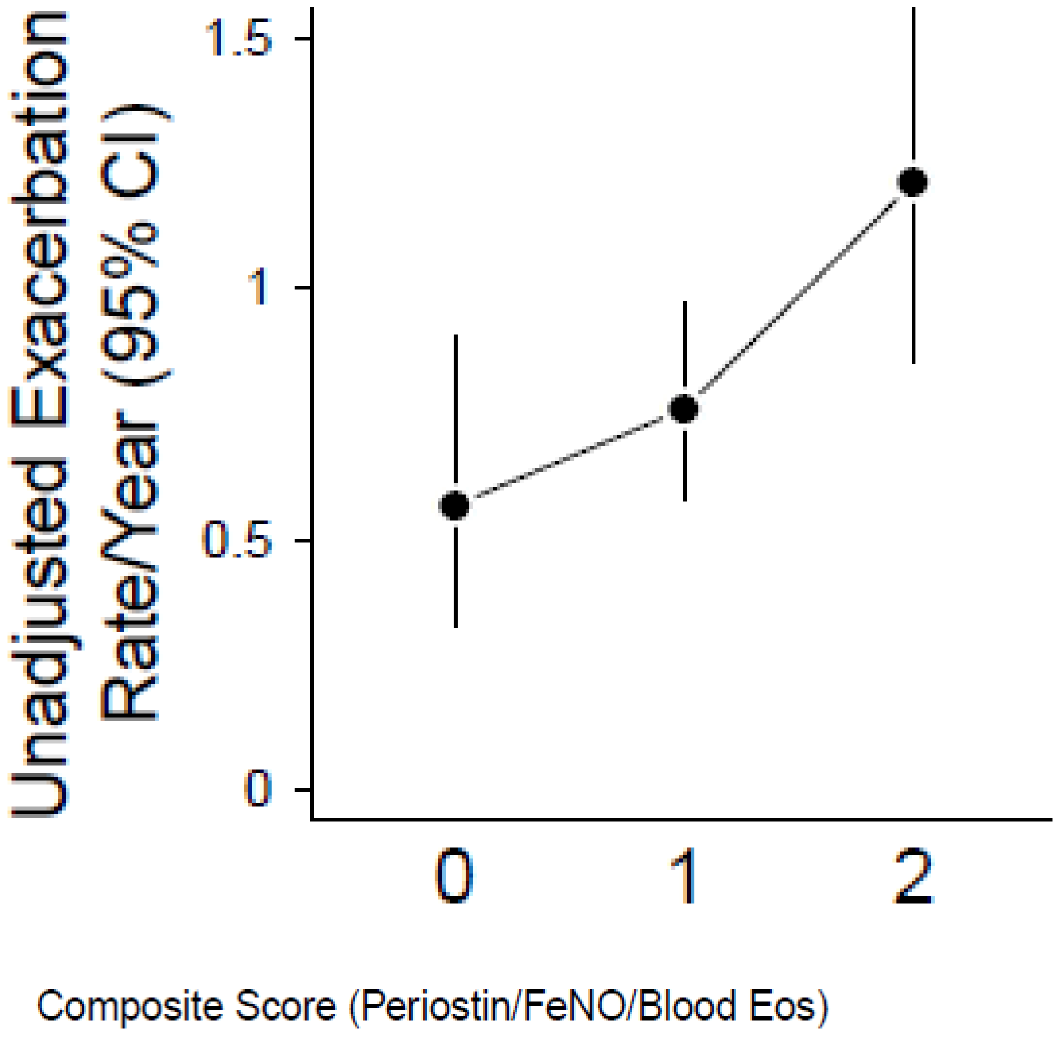
Unadjusted Exacerbation Rate/Year (95% CI)
1.5
1
0.5
0
0
1
2
Composite Score (Periostin/FeNO/Blood Eos)

Figure E2

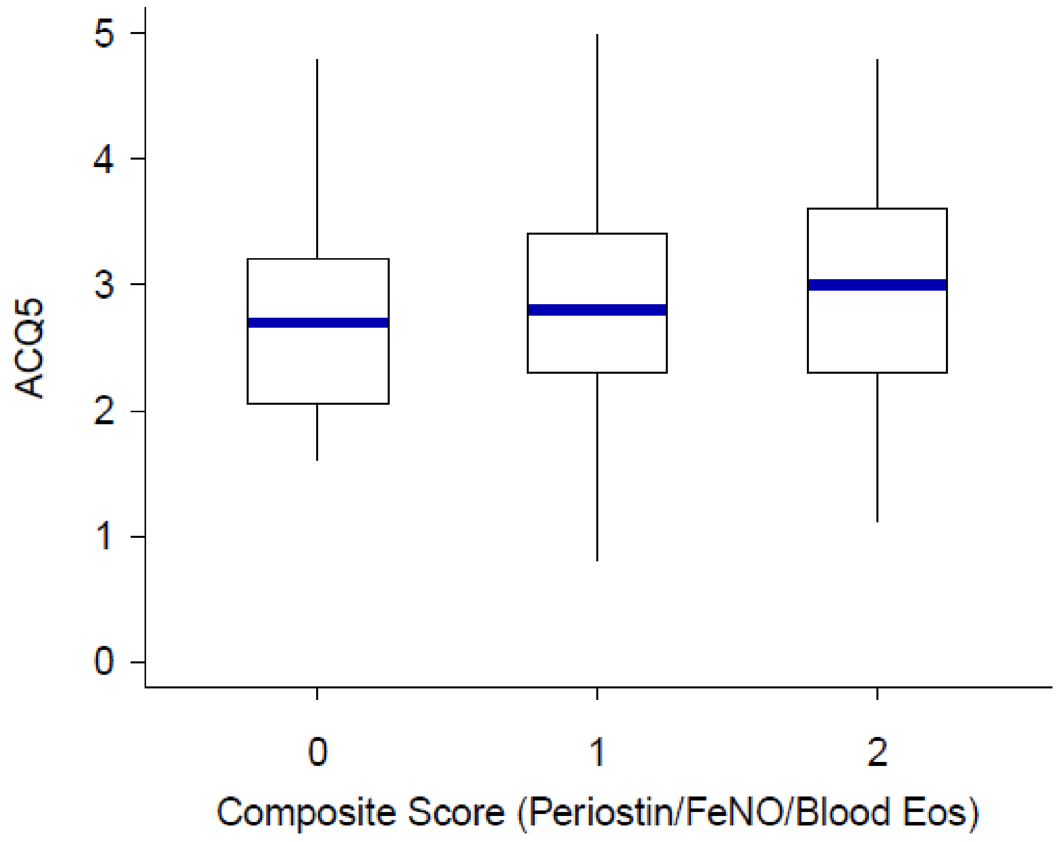

Figure E3

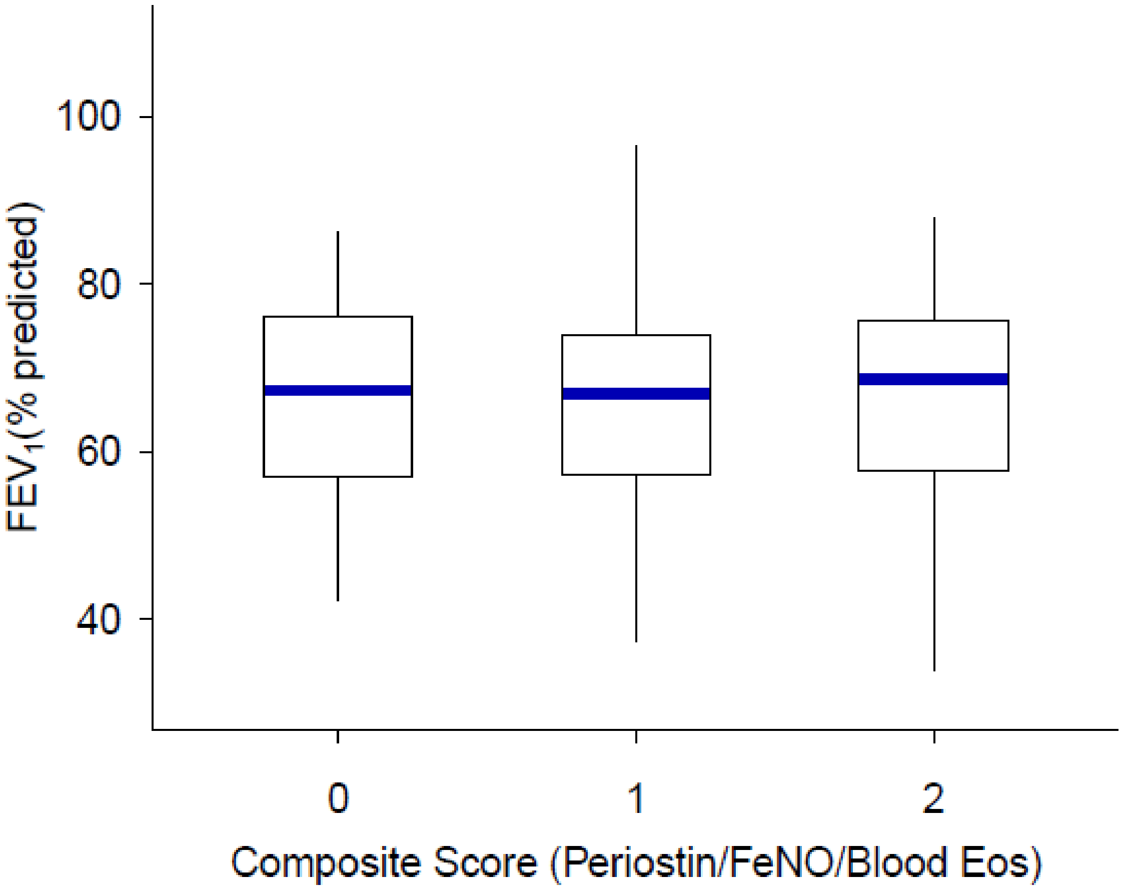

Supplement: Web supplement [file thoraxjnl-2015-207326-s1.pdf]
